# External validation of the preoperative risk evaluation for partial nephrectomy (PREP) score

**DOI:** 10.1002/bco2.70239

**Published:** 2026-07-07

**Authors:** Jacob O'Hara, Kennedy Okhawere, Nicolas A. Soputro, Jewel Bamby, Laurence Hou, Ruben Sauer Calvo, Reza Mehrazin, Ronney Abaza, Simone Crivellaro, Srinivas Vourganti, Ithaar Derweesh, Riccardo Autorino, Craig Rogers, Nitin Yerram, Jihad Kaouk, Mutahar Ahmed, Ketan Badani, Michael Stifelman

**Affiliations:** ^1^ Department of Urology Hackensack University Medical Center Hackensack New Jersey USA; ^2^ Department of Urology Ichan School of Medicine at Mount Sinai New York New York USA; ^3^ Cleveland Clinic Glickman Urological Institute Cleveland Ohio USA; ^4^ Department of Urology University of Illinois at Chicago Chicago Illinois USA; ^5^ Central Ohio Urology Group Dublin Ohio USA; ^6^ Department of Urology Rush University Medical Center Chicago Illinois USA; ^7^ Department of Urology Henry Ford Health System Detroit Michigan USA; ^8^ Department of Urology Hackensack Meridian School of Medicine Nutley New Jersey USA

**Keywords:** complications, multiport, partial nephrectomy, robotic surgery, single port

## Abstract

**Introduction:**

This study aims to externally validate the preoperative risk evaluation for partial nephrectomy (PREP) score, recently introduced to predict the risk of major complications after partial nephrectomy based on patient characteristics, in a large multi‐institutional cohort of robot‐assisted partial nephrectomy (RAPN) patients.

**Materials and Methods:**

A retrospective review was performed on the IRB‐approved, multi‐institutional United States Kidney Cancer Data Network (US‐KIDNET) to identify 10 154 patients who underwent RAPN from 2018 to 2025. Major complications were defined as Clavien–Dindo grade III–V within 30 days. Patients were assigned to risk categories for major complications based on weighted comorbidities, as defined by the PREP score, and the alignment of predicted complication rates with actual complication rates was evaluated. Discrimination was evaluated using the area under the receiver operating characteristic curve (AUC). Calibration was examined using the Hosmer–Lemeshow (HL) goodness‐of‐fit test. Subgroup analysis was performed by robotic platform and surgical approach. Exploratory univariate and multivariate logistic regression were performed to evaluate associations between individual PREP components and major complication rates. All computations were performed in Python (v3.8).

**Results:**

A total of 8615 patients who underwent RAPN were included in the final validation cohort. The PREP score did not perform well in this robotic surgery cohort, with limited overall discrimination (AUC 0.547; 95% CI 0.518–0.580). On calibration, the model's predicted probabilities were not well‐aligned with observed outcomes (HL χ^2^ = 150.2; *p* < 0.001). Performance did not differ significantly across robotic platform or surgical approach subgroups. In the adjusted analysis, coronary artery disease (CAD, aOR 2.22), chronic obstructive pulmonary disease (COPD, aOR 2.75), chronic kidney disease (CKD, aOR 1.48), and obesity (aOR 1.73) were independently associated with major complications, but not congestive heart failure (CHF; aOR 0.50, *p* = 0.36).

**Conclusion:**

Our real‐world external validation for the PREP score demonstrates that this score does not perform well in a large, multicentre, robotic cohort. Our findings suggest that coefficient re‐estimation, rather than simple recalibration, may be required.

## 1 INTRODUCTION

Partial nephrectomy is the standard of care for renal tumours that are technically feasible to remove surgically.^1^ Although there is evidence that the rising implementation of robot‐assisted partial nephrectomy (RAPN) has led to a reduction in perioperative morbidity,^2^, ^3^ RAPN remains a relatively morbid operation, with a complication rate as high as 30%.^1^ As a result, attempts have been made to identify what factors can predict patients who will have poor outcomes after RAPN.

Earlier attempts examined tumour characteristics as the culprit, with validated metrics, such as the R.E.N.A.L nephrometry score^4^ and Preoperative Aspects and Dimensions Used for an Anatomical (PADUA) score,^5^ having conflicting evidence of importance for predicting postoperative complications.^6^, ^7^, ^8^ In fact, there is evidence that the added complexity of these scores adds no greater predictive value than tumour size alone.^9^ Instead, more patient‐specific factors have appeared to have a greater impact. For example, the UroCCR‐57 study developed a nomogram for complication prediction after identifying Charlson Comorbidity Index (CCI) and Eastern Cooperative Oncology Group (ECOG) performance status as most predictive of partial nephrectomy complications in a large robotic cohort.^1^

In 2020, Huynh et al. published the patient factors predicting complications after partial nephrectomy (PREP score).^10^ This nomogram was intended to build upon these attempts at examining the role of patient factors and providing surgeons with more actionable insights for predicting which patients would experience major complications after PN. Huynh et al. demonstrated, using a large public database based on administrative data, that specific comorbidities, chief among them a history of CHF, could effectively predict the incidence of major complications after open, laparoscopic, and robotic partial nephrectomy.

The aim of the present study was to externally validate the PREP score in a purely robotic surgery cohort. By doing so with a large multi‐institutional database composed of data from high volume surgical centres, we hope to provide greater insight to this nomogram in a real‐world context. In addition, we seek to build on this work by examining the model's performance by robotic platform and surgical approaches, as well as with a sensitivity analysis that excludes more complex cases. Finally, given the score's dependence on accurate variable weighting, we performed an exploratory logistic regression to evaluate how each individual PREP score component performed as a predictor of major complications in our own cohort.

## 2 MATERIALS AND METHODS

### 2.1 Study design and endpoint

A retrospective review was performed on the IRB‐approved, multi‐institutional United States Kidney Cancer Data Network (US‐KIDNET) to identify 10 154 patients who underwent RAPN from 2018 to 2025. Data collection at each site was performed using manual chart review with data inputted to site‐specific databases. De‐identified data from each site was then transferred to the host institution maintaining US‐KIDNET. Major complications were defined as Clavien–Dindo grade III–V within 30 days. Patients were assigned to risk categories for major complications based on weighted comorbidities as established in Huynh et al.^10^ (Table 1). The primary endpoint was any major postoperative complication within 30 days of surgery, defined as Clavien–Dindo grade III–V.

**TABLE 1:** Cohort characteristics.

Cohort characteristics	*N* = 8615
Age (years), median (IQR)	61.0 (52–69)
Male sex, *n* (%)	5362 (62.6)
BMI (kg/m^2^), median (IQR)	27.0 (24.1–30.7)
Comorbidities, *n* (%)	
CKD	1141 (13.2)
CHF	91 (1.1)
CAD	339 (3.9)
COPD	298 (3.5)
HTN	4071 (47.3)
DM	1428 (16.6)
Smoker	1208 (17.3)
Obesity	374 (4.3)
PREP score, median (IQR)	8.0 (3.0–12.0)
Risk category distribution, *n* (%)	
Low	5815 (67.5)
Intermediate	2231 (25.9)
High	479 (5.6)
Very high	90 (1.0)
Major complications, *n* (%)	289 (3.4)
Robotic platforms, *n* (%)	
Multiport	8079 (93.8)
Single port	536 (6.2)

### 2.2 PREP score components

The PREP score consists of 10 weighted patient‐specific factors, which are used to calculate a composite score that places the patient in a risk category. The patient characteristics and corresponding points are as follows: Congestive heart failure (CHF) (19 points), chronic kidney disease (CKD) (9 points), coronary artery disease (CAD) (7 points), chronic obstructive pulmonary disease (COPD) (3 points), body mass index (BMI) > =30 (3 points), male sex (3 points), diabetes mellitus (DM) (3 points), smoking (3 points), hypertension (HTN) (2 points), age 65–74 years (5 points), and age > = 75 years (8 points).

A patient's aggregate points based on these comorbidities determine their risk category for major postoperative complications. Low risk patients (≤10 points) had a major complication risk of 3% (95% CI 2.6%–3.2%); intermediate risk patients (11–20 points) of 8% (95% CI 7.2%–9.2%); high risk patients (21–30 points) of 24% (95% CI 20.5%–27.8%); lastly, very high‐risk patients (>30 points) of 41% (95% CI 34.5%–47.8%).

### 2.3 Model evaluation strategy

Three domains of model performance were assessed: discrimination, calibration, and sensitivity.

1. Discrimination: Model discrimination was evaluated using the area under the receiver operating characteristic curve (AUC) with 95% confidence intervals estimated via 1000 bootstrap resamples. The optimal probability threshold was identified using Youden's J statistic, and diagnostic performance (sensitivity, specificity, positive predictive value [PPV], and negative predictive value [NPV]) was reported at the PREP‐defined risk thresholds.
2. Calibration: Calibration was examined using the Hosmer–Lemeshow (HL) goodness‐of‐fit test, Brier score, and comparison between observed and expected complication rates across risk categories. Calibration plots were used to visualise the agreement between predicted probabilities and actual outcomes.
3. Additional Analyses: Subgroup analyses were performed by surgical approach (transperitoneal vs retroperitoneal) and robotic platform (multiport vs single port). A sensitivity analysis was also conducted excluding complex cases such as bilateral tumours, conversions to open or radical nephrectomy, and prior abdominal surgery.

To determine whether available cohort‐level variables were associated with major postoperative complications, logistic regression analyses were performed. Univariate logistic regression was first conducted for demographic, comorbidity, and surgical platform variables. Multivariate logistic regression was then performed in two stages. Model 1 included age, sex, CKD, CHD, CAD, COPD, HTN, DM, current smoking status, and obesity. Model 2 added single‐port and multiport robotic platforms. Results were reported as odds ratios (ORs) with 95% confidence intervals (CIs) and *p*‐values.

All computations were performed in Python (v3.8) using the scikit‐learn, SciPy, and pandas libraries. Statistical significance was defined as *p* < 0.05.

## 3 RESULTS

### 3.1 Cohort characteristics

A total of 8615 patients who underwent RAPN were included in the final validation cohort. The median age was 61 (IQR 52, 69) years, and the median BMI was 27.0 (24.1, 30.7) kg/m^2^. Overall, 289 patients (3.4%) experienced a major complication (Clavien–Dindo Grade III+). The median PREP score was 8.0 (3.0, 12.0). The distribution of patients across PREP risk categories was Low: 5815 patients (67.5%); Intermediate: 2231 patients (25.9%); High: 479 patients (5.6%); and Very High: 90 patients (1.0%) (see Table 1).

### 3.2 Discrimination

**FIGURE 1:**
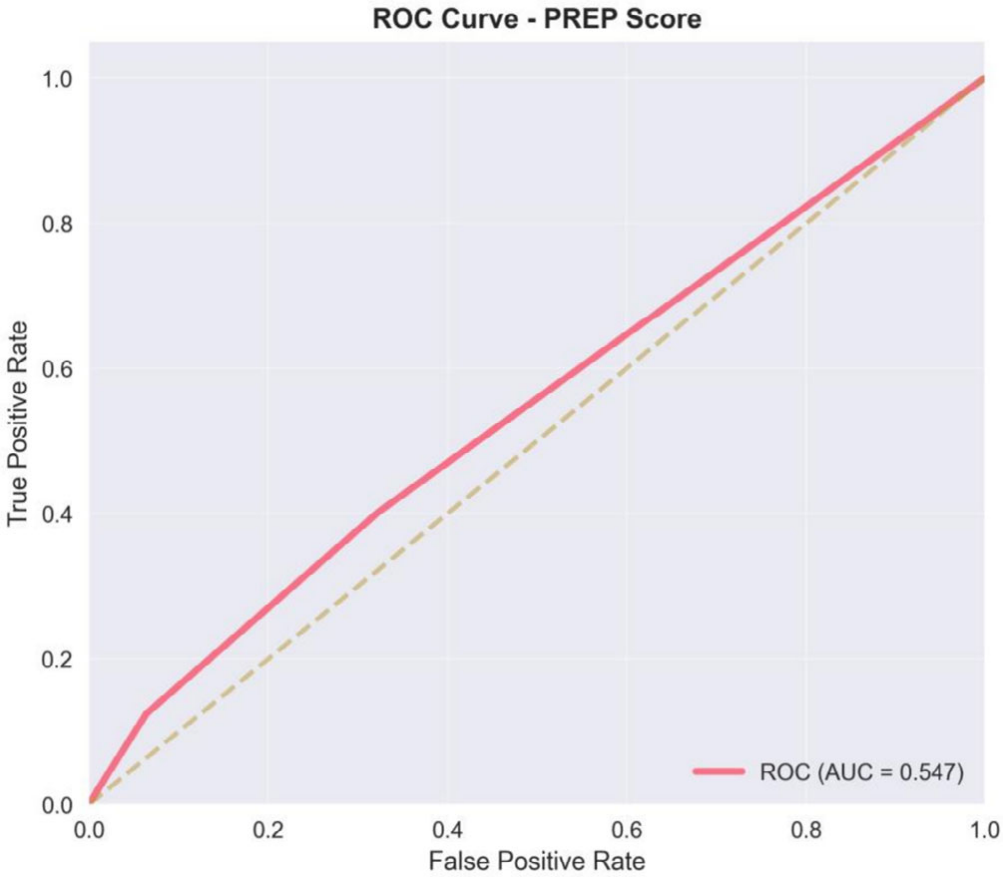
The receiver operating characteristic (ROC) curve for the PREP score. The dashed line represents no predictive value.

The PREP model achieved an AUC of 0.547 (95% CI: 0.518–0.579), indicating limited ability to differentiate between patients with and without major complications. At the optimal probability threshold (Youden's index = 0.08), the model showed a sensitivity of 0.40 and specificity of 0.68. Because major complications were uncommon, the positive predictive value was low (PPV of 0.04), whereas the negative predictive value remained high (NPV of 0.97). At the optimal threshold, the positive likelihood ratio was 1.245, and the negative likelihood ratio was 0.883 (see Figure 1).

### 3.3 Calibration

**FIGURE 2:**
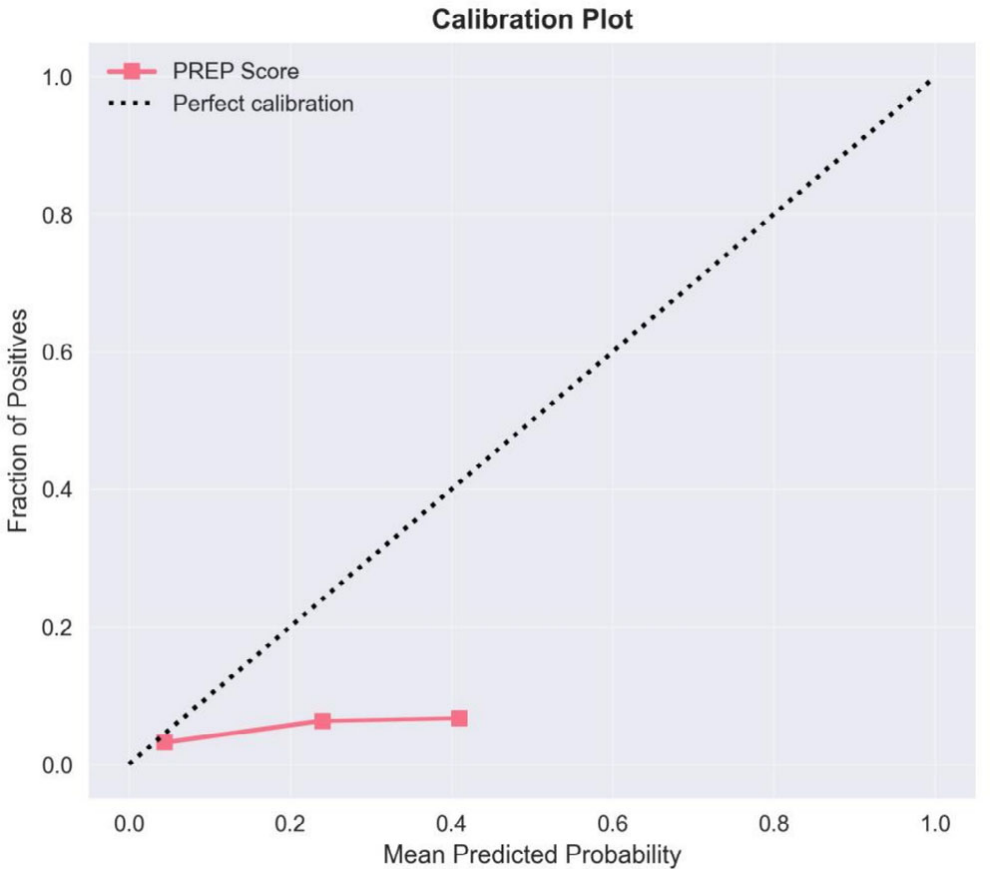
Calibration plot for the PREP score. PREP, preoperative risk evaluation for partial nephrectomy.

The HL test yielded a *χ* ^2^ value of 150.2 (*p* < 0.001), confirming that the model's predicted probabilities were not well aligned with observed outcomes. Discrimination remains suboptimal despite a low Brier score (0.036), which is largely driven by the class imbalance and is indicative of the tendency for systemic overestimation of major complication risk for the higher predicted risk level categories (see Table 2 and Figure 2 **)**.

**TABLE 2:** Calibration by risk categories.

Risk category	*N*	Observed rate	Expected rate	Absolute error
Low	5815	0.030	0.03	0.000
Intermediate	2231	0.036	0.08	0.044
High	479	0.063	0.24	0.177
Very high	90	0.067	0.41	0.343

### 3.4 Exploratory logistic regression

In univariable logistic regression, CKD was associated with increased odds of major complication (OR 1.65, 95% CI 1.16–2.35, *p* = 0.005). CAD (OR 2.95, 95% CI 1.71–5.09, *p* < 0.001), COPD (OR 3.21,95% CI 1.78–5.76, *p* < 0.001), hypertension (OR 1.53, 95% CI 1.16–2.02, *p* = 0.003), and obesity (OR 2.13, 95% CI 1.29–3.50, *p* = 0.003) were also associated with increased odds, whereas age, sex, CHF, diabetes, current smoking, and robotic platform were not statistically significant in univariable analysis (Table 3).

**TABLE 3:** Univariable and multivariable logistic regression for major postoperative complications.

Variable	Univariable		Model 1		Model 2	
	OR (95% CL)	*p*‐value	Adjusted OR (95% CL)	*p*‐value	Adjusted OR (95% CL)	*p*‐value
Age at surgery	1.01 (0.99–1.02)	0.308	1.00 (0.99–1.01)	0.659	1.00 (0.99–1.01)	0.662
Male sex	0.75 (0.55–1.01)	0.056	0.74 (0.55–1.01)	0.058	0.74 (0.55–1.01)	0.057
CKD	1.65 (1.16–2.35)	0.005	1.48 (1.01–2.15)	0.042	1.48 (1.01–2.15)	0.042
CHF	1.14 (0.28–4.72)	0.852	0.50 (0.11–2.18)	0.356	0.50 (0.11–2.17)	0.353
CAD	2.95 (1.71–5.09)	<0.001	2.22 (1.25–3.96)	0.007	2.23 (1.25–3.96)	0.006
COPD	3.21 (1.78–5.76)	<0.001	2.75 (1.49–5.08)	0.001	2.74 (1.48–5.07)	0.001
Hypertension	1.53 (1.16–2.02)	0.003	1.33 (0.98–1.81)	0.064	1.33 (0.98–1.81)	0.066
Diabetes	1.16 (0.81–1.66)	0.423	0.91 (0.62–1.34)	0.643	0.91 (0.62–1.34)	0.642
Current smoker	1.07 (0.75–1.53)	0.706	1.09 (0.76–1.57)	0.633	1.09 (0.76–1.57)	0.631
Obesity	2.13 (1.29–3.50)	0.003	1.73 (1.02–2.93)	0.040	1.73 (1.02–2.93)	0.042
Robotic platform						
Single port	Ref				Ref	
Multiport	1.18 (0.72–1.94)	0.500	‐	‐	1.04 (0.63–1.72)	0.874

*Note*: Model 1 included age at surgery, sex, CKD, CHF, CAD, COPD, hypertension, diabetes, current smoking status, and obesity. Model 2 included Model 1 variables plus robotic platform.

Abbreviations: CAD, coronary artery disease; CHF, congestive heart failure; CKD, chronic kidney disease; COPD, chronic obstructive pulmonary disease; CL, confidence limits; OR, odds ratio.

In the adjusted model (Table 3), controlling for age at surgery, sex, CKD, CHF, CAD, COPD, hypertension, diabetes, current smoking status, and obesity, CKD (adjusted OR [aOR] 1.48, 95% CI 1.01–2.15, *p* = 0.042), CAD (aOR 2.22, 95% CI 1.25–3.96, *p* = 0.007), COPD (aOR 2.75, 95% CI 1.49–5.08, *p* = 0.001), and obesity (aOR 1.73, 95% CI 1.02–2.93, *p* = 0.040) remained independently associated with major complications.

Model 2 added robotic platform. The adjusted associations were essentially unchanged after adding robotic platform. CKD (aOR 1.48, 95% CI 1.01–2.15, *p* = 0.042), CAD (aOR 2.23, 95% CI 1.25–3.96, *p* = 0.006), COPD (aOR 2.74, 95% CI 1.48–5.07, *p* = 0.001), and obesity (aOR 1.73, 95% CI 1.02–2.93, *p* = 0.042) remained independently associated with major complication. Robotic platform was not associated with major complication (single‐port vs multiport: aOR 1.04, 95% CI 0.63–1.72, *p* = 0.874).

### 3.5 Subgroup analysis

Model discrimination was consistent across subgroups. When performing model discrimination across robotic platform subgroups, the AUC for multiport (*n* = 8079) RAPN was 0.543 and for single‐port (*n* = 536) was 0.599. For discrimination across surgical approach subgroups, the AUC for transperitoneal (*n* = 4331) RAPN was 0.508 and for retroperitoneal (*n* = 1008) RAPN was 0.573. Performance was slightly better for single‐port and retroperitoneal procedures, but differences were not statistically significant (see Table 4).

**TABLE 4:** Model discrimination by robotic platform and surgical approach subgroups.

Robotic platform	*N*	Major complications, *n* (%)	PREP score, mean	AUC
Multiport	8079	267 (3.3)	8.637	0.543
Single port	536	20 (3.7)	10.071	0.599
Surgical approach	*N*	Major complications, *n* (%)	PREP Score, mean	AUC
Transperitoneal	4331	134 (3.1)	8.397	0.508
Retroperitoneal	1008	37 (3.7)	8.032	0.573

### 3.6 Sensitivity analysis

After removing complex cases (bilateral tumours, conversions, or prior abdominal surgery)

5352 patients remained in the sensitivity cohort. Model performance did not materially change (AUC = 0.521, Brier = 0.035, HL *p* < 0.001), confirming the robustness of the findings.

## 4 DISCUSSION

The prediction of complications following PN has been an evolving goal for many years, and our findings with this external validation of the PREP score warrant interpretation within the context of the literature. Early efforts attempting to determine postoperative risk for complication after partial nephrectomy were often focused on single metrics, especially tumour complexity metrics like the R.E.N.A.L, PADUA, and C‐index nephrometry scores.^4^, ^5^, ^11^ These scores have demonstrated some predictive power for complication rates following partial nephrectomy but typically only modest discrimination with AUC's ranging anywhere from 0.59 to 0.88.^12^, ^13^, ^14^ These results seem to indicate that understanding tumour features alone is not sufficient to make predictions regarding a patient's risk for complications. The failure of these single‐metric nomograms prompted the development of nomograms that incorporated a broader range of variables. Notably, in a prospective multicentre observational study, Mari et al. achieved a similar AUC of 0.61 after incorporating American Society of Anaesthesiologists (ASA) score, tumour staging, PADUA score, preoperative anaemia, and open and laparoscopic PN versus RAPN.^15^ Interestingly, robotic approach emerged as the strongest predictor of reduced postoperative complications after partial nephrectomy, which highlights the importance of considering the heterogeneity of one's surgical population.^15^ At a similar time, Khene et al. achieved an AUC of 0.75 in a large, multi‐institutional, purely robotic cohort for the prediction of major postoperative complications with a nomogram incorporating male sex, Charlson Comorbidity Index (CCI), ECOG performance status (PS), hospital volume, and R.E.N.A.L nephrometry score.^1^ Of note, the authors reported that CCI and ECOG PS were the strongest predictors for major complication after partial nephrectomy and that these findings were in line with another study, which found age >75 and CCI > 2 to be the most accurate predictors.^1^, ^16^ This trajectory of the literature served as the foundation for the PREP score, which tested a purely patient‐factor nomogram in a cohort of open, laparoscopic, and robotic PN patients and yielded an AUC of 0.73.^10^ Given the relative strength that patient‐factor data showed in a heterogenous cohort with the PREP score and in a purely robotic cohort with the UroCCR‐57 study, as well as the importance of robotic approach relative to open and laparoscopic approaches demonstrated in the RECORd 2 trial, our institution determined that the PREP score should be externally validated in our own large multi‐institutional, purely robotic surgical cohort.

Our findings indicate that the PREP score model performs poorly in this real‐world robotic surgery cohort, as indicated by the model's poor discrimination (AUC 0.547) and calibration (HL *p* < 0.001). In addition, the finding of non‐significance on subgroup analysis when comparing single‐port to multiport (AUC 0.599 vs 0.543) and retroperitoneal to transperitoneal (AUC 0.573 vs 0.508) is not surprising based on recent meta‐analyses, demonstrating no significant difference in major complications when comparing RAPN by robotic platform and surgical approach subgroups.^17^, ^18^ Regarding clinical actionability, the PREP score's AUC of 0.547 indicates that the incidence of major complication after RAPN can be predicted only marginally better than chance using the PREP score. The calibration of the PREP score demonstrates progressive overestimation of major complication risk as risk‐category increases. For example, the observed major complication rate for high risk‐category patients in our US‐KIDNET sample was 6.3%, compared to the predicted rate of 24% obtained from the PHD. Surgeons providing patient counselling to those falling in the high‐risk category based on the PREP score may be unnecessarily dissuaded from nephron‐sparing surgery based on an exaggerated estimation of risk.

Our exploratory logistic regression analysis offers further insight into why the PREP score performed poorly in our multi‐institutional robotic cohort. Although the model's poor calibration is partly driven by the low overall major complication rate (3.4%), the variable weighting itself appears to contribute. In the PREP score, CHF is the most heavily weighted comorbidity (19 points) and exceeds the next‐most heavily weighted comorbidity (CKD) by 10 points. In our cohort, however, CHF was not associated with the incidence of major complication in either univariate or adjusted analyses. Conversely, CAD and COPD, which were assigned 7 and 3 points, respectively, in the PREP score, emerged as the strongest independent predictors of major complication. CKD and obesity were also independently associated with major complication rate, while age, sex, hypertension, diabetes, and cigarette smoking did not retain significance in adjusted models despite being weighted in the PREP score. These findings suggest that the PREP score's issues extend beyond simple miscalibration and that the original PREP score weighting may not reflect the relationships between comorbidities and complications found in a contemporary robotic cohort. Re‐weighting of the PREP score, or the development of a new risk model specific for a contemporary robotic cohort, may be more impactful than recalibration of the PREP score alone.

Beyond the variable‐weighting issues identified earlier, several broader factors likely also contribute to the reduced predictive strength of the PREP score on external validation. First, there are notable differences in the surgical approaches evaluated in our external validation compared to the original validation performed by Hyunh et al. The original authors validated the PREP score in a cohort that included open and laparoscopic cases in addition to multiport robotic cases. In contrast, the present study exclusively evaluates partial nephrectomies performed with the multiport or single‐port robotic surgical platforms. This composition is reflective of the shift in urologic surgery towards robotic approaches, especially at high volume centres, but inevitably changes the overall morbidity of our cohort compared to the original cohort, and may have reduced event rates and altered predictor–outcome relationships. Although Huynh et al. does not report the rates of open complications, we can assume they are driving the higher complication rates compared to those seen in US‐KIDNET. On univariate analysis of predictors for major complication following partial nephrectomy, Hyunh et al. found robot‐assisted surgery to be associated with significantly lower risk for major complication relative to open surgery (OR 0.79; 95% CI 0.62–0.98; *p* = 0.035), although this difference was not significant on multivariate analysis (OR 0.84; 95% CI 0.67–1.06; *p* = 0.144).^10^

Second, there are distinct differences between the populations used to evaluate the PREP score. US‐KIDNET is a multi‐institutional database composed of data from high‐volume centres, whereas the Premier Health Database (PHD) employed in the initial validation of the PREP score is a large public database based on administrative claims data. Huynh et al. acknowledges the PHDs reliance on claims data as a limitation to their study, noting that the PHD does not capture specific data that could impact complication rates, such as history of prior abdominal surgery, tumour characteristics, and comorbidity severity.^10^ This is a commonly remarked upon limitation to studies that utilise databases based on claims data or national databases in general. Both the authors of the PREP score as well as other studies using similar databases have also commented on the reliability of Clavien–Dindo classifications for complications, whether it be underreporting, overreporting, or incorrect classification of complications.^10^, ^19^, ^20^ Conversely, such large repositories of data from a wide variety of medical practices offer a great deal of generalisability that may be difficult to achieve otherwise, even at high‐volume regional centres. Third, it is important to note that nomograms rarely perform as well on external validation compared to the population they were trained on. We would therefore expect to see a decline in performance because of differences in the overall makeup of our patient population, although the decline in performance from 0.73 in the initial validation to 0.547 in our external validation is likely too large to be explained by this effect alone.

We acknowledge there are limitations to this study. First, this analysis was performed retrospectively, which could introduce selection bias. Second, the combination of population differences between patients in the PHD and US‐KIDNET as well as the institutions contributing to US‐KIDNET being high‐volume centres could also introduce bias, which could have contributed to the PREP scores' poor performance in our study population compared to the PHD. Third, the possibility of variations in complication reporting between US‐KIDNET and the PHD could contribute to the differences we see in model performance. Similarly, there is no breakdown of individual complications to compare which major complications were more prevalent in the PHD compared to US‐KIDNET. Lastly, the PREP score's exclusion of tumour and surgeon variables further limits its applicability. Combined with the re‐weighting concerns identified in our exploratory analysis, this suggests that future iterations should incorporate case‐complexity variables alongside re‐estimated comorbidity coefficients. Although outside of the current scope of this external validation study, future direction for this research at our institution will include building a more accurate prediction tool based on these more diverse variables.

## 5 CONCLUSION

This first external validation of the PREP score in a large, multi‐institutional, high‐volume cohort demonstrates poor discrimination and calibration, with progressive overestimation of major complication risk in a contemporary robotic surgery cohort. Our exploratory analysis suggests that the PREP score's underperformance reflects not only the miscalibration but also the misspecification of individual variable weights. Coefficient re‐estimation, combined with the addition of tumour‐specific and surgeon‐level variables that are not captured in the PREP score, may represent a more promising path towards an accurate complication risk prediction model for a contemporary robotic partial nephrectomy population.

## AUTHOR CONTRIBUTIONS

**Jacob O'Hara:** Conceptualization; investigation; methodology; writing—original draft; critical revisions; writing—final draft; response to reviewers. **Kennedy Okhawere:** Statistical analysis; writing—original draft; critical revisions; writing—final draft; response to reviewers. **Nicolas A. Soputro:** Writing—review and editing. **Jewel Bamby:** Writing—review and editing. **Laurence Hou:** Writing—review and editing. **Ruben Sauer Calvo:** Writing—review and editing. **Reza Mehrazin:** Writing—review and editing. **Ronney Abaza:** Writing—review and editing. **Simone Crivellaro:** Writing—review and editing. **Srinivas Vourganti:** Writing—review and editing. **Ithaar Derweesh:** Writing—review and editing. **Riccardo Autorino:** Writing—review and editing. **Craig Rogers:** Writing—review and editing. **Nitin Yerram:** Critical revisions; writing—review and editing. **Jihad Kaouk:** Critical revisions; writing—review and editing. **Mutahar Ahmed:** Critical revisions; writing—review and editing. **Ketan Badani:** Critical revisions; writing—review and editing. **Michael Stifelman:** Conceptualization; methodology; supervision; writing—review and editing; critical revisions.

## ACKNOWLEDGEMENTS

The authors have nothing to report.

## CONFLICT OF INTEREST STATEMENT

Neither Jacob O'Hara nor any of the other authors of this manuscript has any conflicts to disclose.
